# Effects of Different Toothpastes on the Nanomechanical Properties and Chemical Composition of Resin-Modified Glass Ionomer Cement and Composite Resin Restorations

**DOI:** 10.3390/dj11070173

**Published:** 2023-07-17

**Authors:** Mariana Dias Moda, Paulo Henrique Dos Santos, Nubia Inocencya Pavesi Pini, Leonardo Negri Furini, André Luiz Fraga Briso, André Assmann, Ticiane Cestari Fagundes

**Affiliations:** 1 Department of Preventive and Restorative Dentistry, Araçatuba School of Dentistry, São Paulo State University (UNESP), Araçatuba 16015-050, SP, Brazil; moda_mariana@hotmail.com (M.D.M.); nubiapini01@gmail.com (N.I.P.P.); andre.briso@unesp.br (A.L.F.B.); 2 Faculty of Dentistry, Dental Research Institute—Restorative Dentistry, University of Toronto, Toronto, ON M5S 1A1, Canada; pauloh.dossantos@utoronto.ca; 3 Department of Physics, Federal University of Santa Catarina, Florianópolis 88040-900, SC, Brazil; 4 Department of Engineering and Exact Sciences, Federal University of Paraná, Palotina 85950-000, PR, Brazil

**Keywords:** abrasion, composite resin, erosion, glass ionomer cement, stannous ion

## Abstract

Purpose: This study evaluates the effects of different toothpastes on the nanohardness and chemical compositions of restorative materials and dental surfaces. Methods: Bovine enamel (*n* = 72) and dentin (*n* = 72) blocks were obtained and restored using RMGIC (*n* = 36) or CR (*n* = 36) to create the following surfaces: dentin adjacent to RMGIC (DRMGIC), enamel adjacent to RMGIC (ERMGIC), dentin adjacent to CR (DCR), and enamel adjacent to CR (ECR). After restoration, one hemiface of each specimen was coated with an acid-resistant varnish to facilitate the creation of control (C) and eroded (E) sides; the latter were achieved by erosion–abrasion cycles as follows: erosion with 1% citric acid: 5 days, four times for 2 min each day; 1% citric acid/abrasion, two times for 15 s, followed by immersion in a toothpaste slurry for 2 min. Toothpastes without fluoride (WF; *n* = 12), with sodium fluoride (NaF; *n* = 12), and with stannous fluoride (SnF_2_; *n* = 12) were used for RMGIC or CR. The specimens were analyzed for nanohardness (H), and chemical composition using energy-dispersive X-ray spectroscopy and Raman microscopy. The data were statistically analyzed using two-way repeated measures ANOVA and Tukey’s test (α = 0.05). Results: Lower H values were obtained with NaF for DRMGIC-C, with a statistically significant difference from the H value obtained with WF (*p* < 0.05). The calcium and phosphorus concentrations in DCR-E were significantly lower with WF than with the other types of toothpaste (*p* < 0.05). Fluoride-containing toothpastes are capable of preserving the main chemical components of the dentin adjacent to the restorative materials under erosive–abrasive conditions.

## 1. Introduction

Erosive tooth wear (ETW) involves multiple factors and has increased in frequency in the past decade. The etiology is related to eating and drinking habits, particularly the high consumption of acidic beverages, and may also be associated with bulimia, anorexia, and gastroesophageal disorders [1,2]. Importantly, ETW results from a combination of constant contact with acids and the mechanical forces from tooth brushing, contributing to the removal of surface tissue that is softened by acids [3].

Other factors may influence the progression of erosion, such as the rate of dissolution of dental substrates, which is influenced by the presence of impurities in the mineral content of the substrates [4]. Thus, numerous studies have investigated erosive dynamics by considering different aspects, such as the composition of eroded dental tissues, distinct in vitro protocols to simulate erosive processes [5,6,7], the action of bioactive particles on eroded tissues [8], and the actions of different toothpastes, rinses, or varnishes with various active ingredients, in order to find ways to minimize tooth loss [9,10]. In addition, caries-like and white spot lesions are also vulnerable to erosion once the substrate is damaged [11]. A study comparing the abrasion levels using different toothpastes and fluoride gels concluded that early carious lesions are more susceptible to erosive–abrasive processes than sound substrates, particularly when highly abrasive toothpastes are used for brushing [11].

However, as the erosive process advances, pronounced dental substrate loss may occur, resulting in dentin exposure that necessitates clinical restorative procedures [5]. Existing information about the effects of erosion–abrasion challenges on the interfaces of dental restorative materials are scarce. The erosive process in restorative materials differs from that in the dental tissues [12,13]. Repeated erosion cycles may affect the mechanical properties of these materials and reduce their longevity [14,15]. Composite resin (CR) and resin-modified glass ionomer cement (RMGIC) are most frequently used for direct restorations. Although RMGIC forms a chemical bond with enamel and dentin and releases fluoride, which may reduce the erosive effects on adjacent dental tissues, studies have shown that this material is more susceptible to degradation than CR [7,16]. Therefore, it is important to compare the effects of different toothpastes on these restorative materials adjacent to dental substrates after erosion–abrasion cycles.

Some studies have evaluated the effects of different toothpastes on dental substrates and restorative materials subjected to erosion [9,10,11]; however, the surfaces at the restorative interfaces have not been analyzed. Thus, it is important that the possible interactions between restorative materials and adjacent dental substrates be assessed, especially after erosion–abrasion cycles. Hence, we evaluate the effects of different toothpastes with different active compounds on the nanomechanical properties and chemical composition of dental and restorative materials after erosion–abrasion cycles.

The null hypotheses were that different types of toothpastes: (1) would not affect the nanohardness of the dental substrates and restorative materials after erosion–abrasion cycles and (2) would not affect the chemical composition of the dental substrates after such cycles.

## 2. Materials and Methods

### 2.1. Experimental Design

Two experimental factors were investigated in vitro, namely toothpastes and surfaces (dental and restorative materials) using the following: (1) types of toothpastes—without fluoride (WF, negative control), sodium fluoride (NaF, positive control), and stannous fluoride (SnF_2_); and (2) types of surfaces (as per the dental surface, restorative material, and condition). ERMGIC-C: enamel adjacent to resin-modified glass ionomer cement on the control side; ECR-C: enamel adjacent to composite resin on the control side; RMGIC-C: resin-modified glass ionomer cement on the control side; CR-C: composite resin on the control side; DRMGIC-C: dentin adjacent to resin-modified glass ionomer cement on the control side; DCR-C: dentin adjacent to composite resin on the control side; ERMGIC-E: enamel adjacent to resin-modified glass ionomer cement on the eroded side; ECR-E: enamel adjacent to composite resin on the eroded side; RMGIC-E: resin-modified glass ionomer cement on the eroded side; CR-E: composite resin on the eroded side; DRMGIC-E: dentin adjacent to resin-modified glass ionomer cement on the eroded side; DCR-E: dentin adjacent to composite resin on the eroded side.

**Figure 1:**
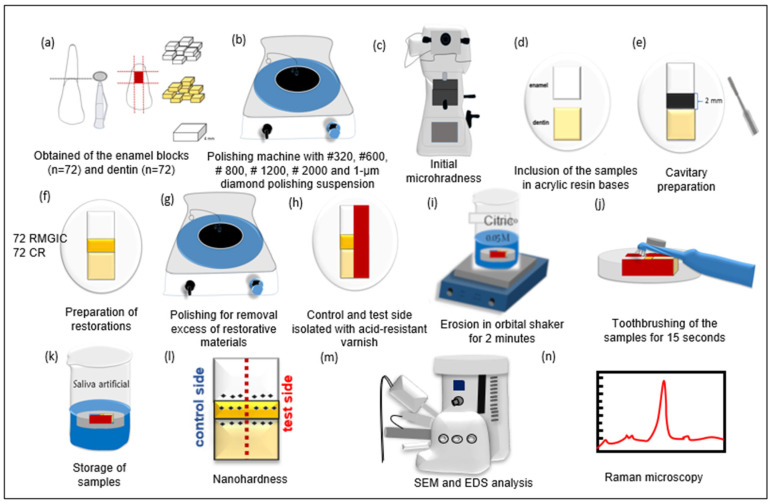
Specimen preparation and study flowchart. (**a**) A total of 144 bovine incisors were obtained and 72 enamel and 72 dentin blocks were created (4 × 4 mm^2^). (**b**) The blocks were then polished in an automatic polishing machine. (**c**) Blocks were selected using a surface microhardness analysis. (**d**) Enamel and dentin blocks were inserted into an acrylic base, 1 mm apart in each base. (**e**) A cavity was prepared on the mesial surfaces of the specimens, with a total surface area of 2 × 2 mm^2^. (**f**) The RMGIC or CR restorations were applied. (**g**) The restorations were polished to remove excess restorative material. (**h**) The hemiface of each specimen/restoration set was covered with an acid-resistant varnish. (**i**) The specimens were subjected to erosion (4 times/day) (**j**) and abrasion (2 times/day) cycles. (**k**) The specimens were stored in artificial saliva between cycles. (**l**) The dental substrates and restorative materials were subjected to H analysis. (**m**) SEM/EDS analyses of the dental surfaces and restorative materials were performed. (**n**) Raman spectroscopy analyses of the dental surfaces were performed.

The characteristics of the toothpastes and restorative materials are listed in Table 1. Figure 1 shows the entire specimen preparation process and the study flowchart.

The evaluated parameters were as follows: nanohardness (H) and all surfaces, chemical composition of the dental surfaces and restorative materials using energy-dispersive X-ray spectroscopy (EDS), and chemical composition of the dental surfaces using Raman spectroscopy.

### 2.2. Specimen Preparation

This study was approved by the local animal ethics committee (process #00243-2018). Bovine incisors were stored in a 0.1% aqueous solution of thymol, for 30 days. A total of 60 enamel and 60 dentin blocks (4 × 4 × 2 mm^2^) were obtained using a precision saw and diamond disk (Isomet 1000; Buehler, Lake Bluff, IL, USA). The samples were then flattened and polished using silicon carbide papers (#320, #600, #1200, and #2000) under constant irrigation and polished using a felt disk with 1 μm diamond paste (Arotec, Cotia, Brazil). The blocks were sonicated in distilled water for 15 min to remove debris. These procedures yielded 1 mm thick enamel and dentin blocks. The Knoop microhardness of these blocks was then analyzed (Micromet 5114; OminiMet Software, Buehler, Lake Bluff, IL, USA) to ensure standardized samples with an enamel hardness of 320–360 KHN and a dentin hardness of 50–70 KHN. All blocks were stored at 100% humidity until use.

### 2.3. Restorative Procedures

Two blocks (one dentin and one enamel) were embedded in an acrylic resin base using a metal matrix, with a distance of 1 mm between the two blocks to facilitate the restorative procedures [17]. A cavity was prepared in the center of the samples using a diamond tip #1090 (KG Sorensen, Barueri, Brazil) that was operated at a high rotational speed and replaced after every fifth preparation. When the preparation was complete, the box-shaped cavity measured 2 × 2 mm [2]. Samples restored with CR were previously conditioned with 37% phosphoric acid for 20 s. Both cavities were filled with their respective restorative materials according to the manufacturer’s instructions and subsequently covered with a polyester strip. A glass slide was placed over the strip and a static load of 0.53 kg was applied using a heavy glass slab to allow excess material to extrude over the top of the cavity margins; this ensured that the material was flush with the surface of the enamel and dentin [14]. Next, the glass slab was removed and the materials were photocured through a polyester strip and glass slide using a light-curing unit with an irradiance of 1000 mW/cm^2^ (Kavo, Joinville, Brazil). Fifty samples were restored using CR (Filtek Z350 XT; 3M ESPE, St. Paul, MN, USA) and photocured for 20 s using a wave LED device (Kavo, Joinville, Brazil). Fifty other samples were restored using RMGIC (Fuji II LC, GC, Tokyo, Japan), photocured for 40 s, and protected with petroleum jelly.

All specimens were kept under humid conditions at 37 °C for 7 days, following which they were polished, as previously described, to remove excess material (#800, #1200, #2000, and felt disk). The hemiface of each specimen was protected, using an acid-resistant varnish (Colorama; São Paulo, Brazil), to create the control and eroded sides [14].

Subsequently, the specimens were randomly assigned to three experimental groups: (1) WF: without fluoride (Curaprox Enzycal Zero; Trybol, Neuhausen am Rheinfall, Switzerland), (2) NaF: sodium fluoride (Colgate total 12; Palmolive, São Bernardo do Campo, Brazil), and (3) SnF_2_: stannous fluoride (Crest Pro-Health; Procter & Gamble, Cincinnati, OH, USA).

### 2.4. Erosion–Abrasion Cycles

The specimens were subjected to erosion–abrasion cycles for 5 days. Erosion cycles were performed 4 times each day, and abrasion cycles were applied after the first and last erosion cycles, daily. The samples were eroded by immersion in 250 mL of 1% citric acid (Merck; Darmstadt, Germany, pH = 3.2) for 2 min under agitation in an orbital shaking table (Tecnal TE–420; Piracicaba, Brazil) at 70 rpm. Toothpaste slurries (WF, NaF, and SnF_2_) were prepared with distilled water (1:3), and 2 mL of this solution was pipetted onto the samples after the first and last erosion cycles; this was followed by abrasion with an electric toothbrush, which weighed 200 g, using a circular motion (Oral-B Plak Control Ultra; Braun, Frankfurt, Germany) for 15 s and subsequent immersion in the slurry for 2 min [10]. Each daily challenge was performed within a 1 h interval, and the samples were stored at 37 °C in artificial saliva (1.5 mmol·L^−1^ Ca(NO_3_)_2_·4H_2_O; 0.9 mmol·L^−1^ NaH_2_PO_4_·2H_2_O; 150 mmol·L^−1^ KCl, 0.1 mmol·L^−1^ buffer Tris; 0.03 ppm F; pH 7.0, Aphoticario, Araçatuba, Brazil) [18]. At the end of the experimental period, the acid-resistant layer was removed and the samples were stored at 100% humidity.

### 2.5. Analyses of Nanohardness (H)

Nanohardness was measured using a nanohardness tester (UNAT; ASMEC, Zwick-Roell, Ulm, Germany). A Berkovich diamond tip was used at a load of 1000 µN and a standard trapezoidal load function of 5–2–5 s [19]. Three measurements were obtained from each of the following regions on each specimen: control and eroded dental substrates adjacent to the restorative interface, RMGIC, and CR at the center of the restoration. In total, there were 18 indentations for each specimen. H was calculated from the load–displacement curves using the following formulae [20].

(1) H=Pmax/A

where Pmax is the maximum load and A is the projected contact area between the indenter tip and sample under maximum load.

### 2.6. Energy-Dispersive X-ray Spectroscopy (EDS) and Scanning Electron Microscopy (SEM)

The surface compositions of the dental substrates and restorative materials were analyzed using by EDS and SEM (EVO LS 15; Carl Zeiss, Oberkochen, Germany) and coated with gold using the Q150T coater (Quorum Technologies, Laughton, UK). Three specimens from each group were selected for EDS analysis of the control and eroded surfaces of the dental substrates and restorative materials using INCAx-act (Oxford Instruments, Concorde, NH, USA) over a defined area of 200 × 200 μm^2^; an electronic mode (20 kV) with 2000x magnification was used. A representative image of all groups was also obtained by SEM at 2000x and 5000x magnifications [21].

### 2.7. Micro-Raman Spectroscopy

Raman measurements were performed using a micro-Raman spectrometer (Renishaw, in-Via model, London, UK) equipped with a CCD detector. The laser was applied at 785 nm with a diffraction grating of 1200 lines/mm. Spectra were recorded with an exposure time of 10 s and one accumulation. Optical images were obtained using a 50x objective lens [22]. Analyses were performed using the integrated areas of the Raman peaks attributed to phosphate and carbonate groups at 960 and 1070 cm^−1^, respectively [23].

### 2.8. Statistical Analysis

All statistical analyses were performed using the SigmaPlot version 12.5 software (Systat Software, San José, CA, USA). The data were analyzed for normality using the Shapiro–Wilk test. H values and EDS data for the dental surfaces and restorative materials were analyzed using two-way repeated measures ANOVA and Tukey’s post hoc test. The enamel, dentin, and restorative materials were considered separately. Raman-integrated area peak values for the dental surfaces only were subjected to two-way repeated measures ANOVA and Tukey’s post hoc test. The level of significance was set at α = 0.05.

## 3. Results

### 3.1. Nanohardness (H)

The nanohardness values are listed in Table 2. There were significant differences only for DRMGIC-C, with a lower H value for the NaF group than for the WF group (*p* = 0.03). When the control surfaces treated with the same toothpaste were compared, there were significant differences between ERMGIC-C and ECR-C treated with WF (*p* = 0.04) and between RMGIC-C and CR-C treated with all toothpaste types (*p* < 0.05). Differences between restorative materials were also observed for the eroded surfaces. Only surfaces that were altered after erosion–abrasion cycles had RMGIC-E for NaF toothpaste and eroded CR-E for all types of toothpaste (*p* > 0.05).

### 3.2. Energy-Dispersive Spectroscopy (EDS)

The results of EDS analyses of the enamel and dentin surfaces are presented in Table 3 and Table 4, respectively. The calcium/phosphorus (Ca/P) ratio for the enamel surfaces showed no significant differences according to the toothpaste type or the challenge (control or erosion) condition (*p* > 0.05). However, no statistical analysis was performed for these elements because they were not observed in any of the specimens in the study. In contrast, the Ca/P ratio for DCR-E showed a significant difference, with a lower value with WF than with NaF (*p* = 0.003). When dentin surfaces were treated with WF and SnF_2_, there were significant differences for the eroded surfaces, presenting lower values than those for the control surfaces (*p* < 0.05).

### 3.3. Analysis of Micro-Raman Spectroscopy

**Figure 2:**
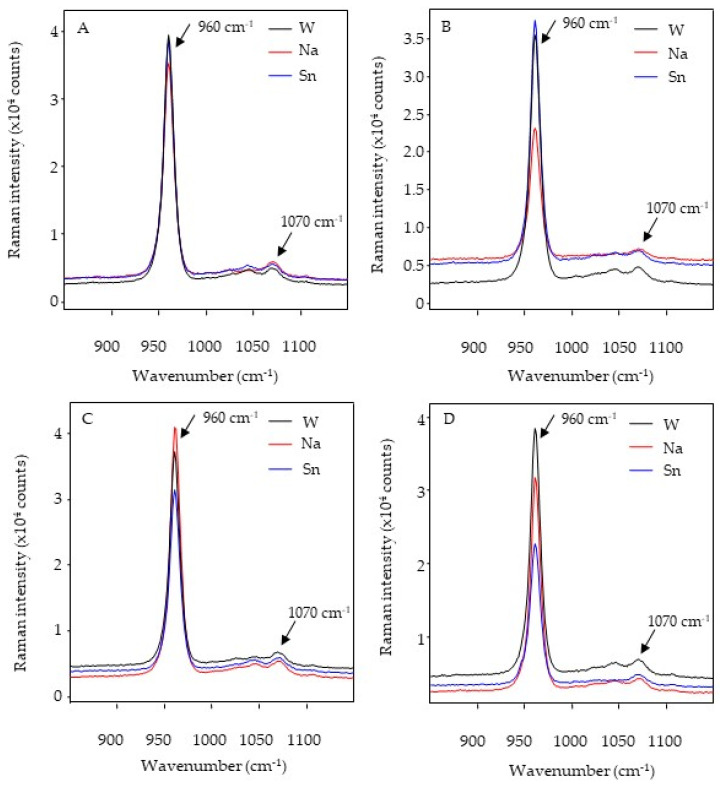
Raman spectroscopy for the control and eroded enamel, with a phosphate peak at 960 cm^−1^ and carbonate peak at 1070 cm^−1^. (**A**) ERMGIC-C, (**B**) ERMGIC-E, (**C**) ECR-C, and (**D**) ECR-E.

**Figure 3:**
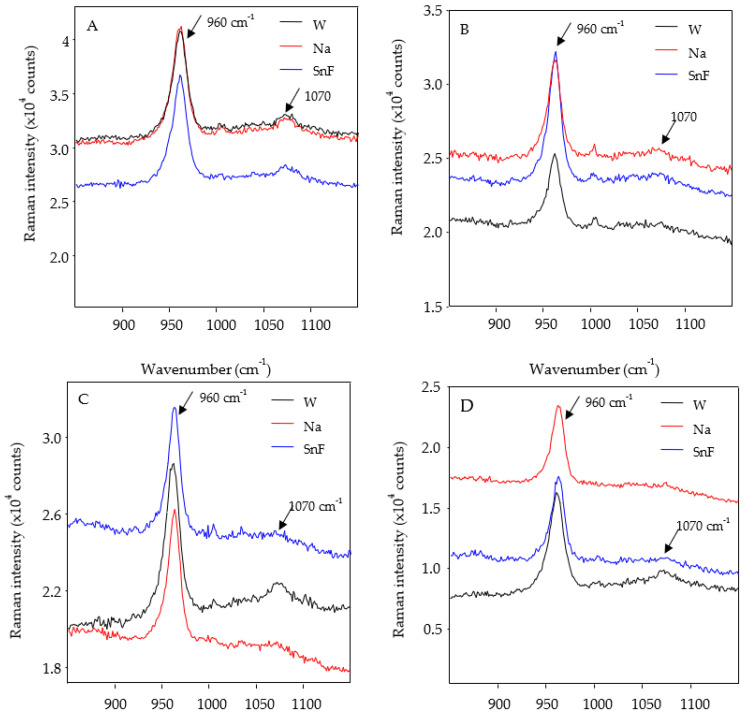
Raman spectroscopy for the control and eroded dentin, with a phosphate peak at 960 cm^−1^ and carbonate peak at 1070 cm^−1^. (**A**) DRMGIC-C, (**B**) DRMGIC-E, (**C**) DCR-C, and (**D**) DCR-E.

The common peaks and areas detected in both enamel and dentin were phosphate (960 cm^−1^) and carbonate (1070 cm^−1^) in the Raman analysis (Figure 2 and Figure 3).

There were no significant differences in relation to the phosphate and carbonate areas among the toothpaste types for the enamel surfaces (*p* > 0.05). When surfaces were compared for a single toothpaste, ECR-E presented a lower phosphate area with SnF_2_ (*p* < 0.05) and ERMGIC-C presented a lower carbonate area with NaF (*p* < 0.05) (Table 5). With regard to phosphate areas on dentin surfaces, SnF_2_ was associated with higher area values than NaF (*p* < 0.05). When dentin surfaces treated with the same toothpaste were compared, the control surfaces (DRMGIC and DCR) presented higher phosphate and carbonate area values than the eroded surfaces (DRMGIC and DCR) for each toothpaste type (*p* < 0.05) (Table 6).

### 3.4. Scanning Electron Microscopy (SEM)

**Figure 4:**
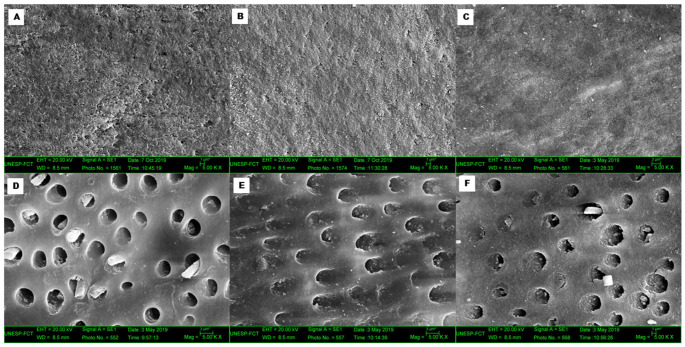

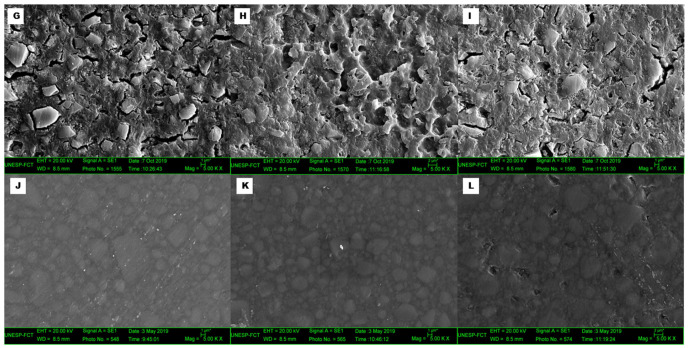
Representative SEM images of eroded surfaces (5000×). (**A**) Eroded enamel surface brushed with WF toothpaste shows roughness. (**B**) Eroded enamel surface brushed with NaF toothpaste shows roughness. (**C**) Eroded enamel surface brushed with SnF_2_ toothpaste shows mineral precipitation. (**D**) Eroded dentin surface brushed with WF toothpaste shows large dentinal tubules and presence of odontoblast processes. (**E**) Eroded dentin surface brushed with NaF toothpaste shows partial obliteration of dentinal tubules. (**F**) Eroded dentin surface brushed with SnF_2_ also shows partial obliteration of dentinal tubules. (**G**) RMGIC-E surface brushed with WF toothpaste shows some cracks. (**H**) RMGIC-E surface brushed with NaF toothpaste shows irregularities. (**I**) RMGIC-E surface brushed with SnF_2_ toothpaste shows cracks and concavities. (**J**) CR-E surface brushed with WF toothpaste shows no alterations. (**K**) CR-E surface brushed with NaF toothpaste shows no alterations. (**L**) CR-E surface brushed with SnF_2_ shows grooves.

Representative SEM images are shown in Figure 4. All eroded surfaces showed differences from the control surfaces; therefore, only images of the eroded surfaces are presented. With regard to the eroded enamel surfaces (Figure 4A–C), there were few differences among the various toothpaste types. However, SnF_2_ (Figure 4C) showed mineral precipitate formation. With regard to the eroded dentin surfaces, in addition to the differences found between the control and eroded surfaces, larger dentinal tubules were observed in the WF group (Figure 4D), whereas mineral precipitate formation of dentinal tubules was observed in the NaF and SnF_2_ groups (Figure 4E and 4F, respectively). Considerable alterations were found on the erosive surfaces for RMGIC (Figure 4G–I), irrespective of the toothpaste type. The CR-E surfaces showed minimal morphological alterations after WF and NaF treatment (Figure 4J,K). However, SnF_2_ (Figure 4L) resulted in a grooved surface.

## 4. Discussion

Hardness analysis is one of the most widely used quantitative methods for measuring the mechanical properties of dental substrates and restorative materials [24]. There are distinct types of hardness depending on the indenter type, load, and penetration depth [24]. Depending on the substrate to be analyzed and the degree of tissue erosion, the measurement of the surface microhardness may be inaccurate or impossible because the indentation limits are unclear [24]. Hardness measurement also requires small indentations, enabling the differentiation of intertubular, peritubular, and dentinal–tubular areas [25]. In the present study, dentin indentations were performed in the intertubular region. It is possible to analyze both elastic deformation, which is transient, and plastic deformation, which is permanent [20,24].

The first null hypothesis of the present study was rejected because there were differences in the nanohardness property. One reason for the differences in H values for the control DRMGIC surfaces could be the diffusion of citric acid or toothpaste slurry through the control surface, which was isolated by acid-resistant varnish. Moreover, the blocks were previously standardized on the basis of the surface Knoop microhardness. This effect was observed in a previous study [26].

With regard to hardness, an interesting finding in the present study was that, although there was no significant difference among the enamel surfaces treated with different toothpastes, enamel surfaces abraded with SnF_2_-based toothpaste showed a hardness reduction of approximately 50% compared to that with other toothpastes. This reduction may be associated with the binding between negative zeta potentials of abrasive silica particles and positive stannous ions (Sn^2+^), which may reduce the anti-erosive action of the toothpaste [9]. In this study, regardless of the toothpaste used for treatment, all eroded surfaces except those restored with CR showed a decrease in H values. Thus, no toothpaste was able to maintain the nanomechanical properties, possibly because a protective layer did not form. SEM images (Figure 4A–C) showed enamel surfaces with notable irregularities and the absence of a significant protective layer. A study that evaluated the application of NaF and titanium tetrafluoride (TiF_4_) varnishes concluded that NaF was not able to form a protective layer on enamel [27]. Moreover, H values may be affected by factors such as the region of indentation [25]. It is worth noting that there were no differences among these toothpaste types in another study by our group, where ultra-microhardness was used to evaluate dentin surfaces [26].

With regard to restorative materials, hardness tests allow an indirect evaluation of the degree of monomer conversion to polymers (a material with higher hardness values has a better polymerization conversion rate) [28,29]. In general, RMGIC is vulnerable to erosion, with a decrease in H values. Therefore, it is important to highlight that indentations on the RMGIC surfaces were placed in the area containing the polymeric matrix instead of that containing inorganic particles. The ionomeric material naturally has a lower hardness than CR, as observed in a previous study [26]. In addition, the association of the erosive process with abrasion using toothpastes with different abrasive levels seems to have accentuated the changes in the structure of RMGIC and contributed to the decrease in its mechanical values [30], which was more notable after brushing with an SnF_2_-based toothpaste. Conversely, CR presented higher values than RMGIC, in terms of mechanical properties, consistent with the findings in another study [30]. In addition, the erosion–abrasion cycles had no effect on CR restorations, independent of the toothpaste used. This is likely associated with the composition of the organic matrix (Bis-GMA) and the arrangement or percentage of nanoparticles [31]. Although the nanohardness used in this study is well established in the literature, a study using the Hertzian indentation applied to GIC showed that this test would be well suited to restorative materials, since it is possible to change the indenter size, as well as the thickness of the sample. In addition, the authors showed that this type of mechanical test reproduces tooth cusp compression of dental restoratives under mastication, which represents the behavior of different types of restorative materials, especially in vitro studies, with clinical relevance [32].

When the same type of surfaces brushed with the same type of toothpastes were compared, superior mechanical properties were observed for ERMGIC than for ECR treated with WF, with the exception of the H value for eroded enamel. The fluoride released only from RMGIC may act on the enamel surface when a fluoride-free toothpaste is used for toothbrushing [33].

The second null hypothesis was also rejected because there were differences in the chemical composition of the dental surfaces and restorative materials. EDS is widely used to investigate the chemical composition of surfaces; it uses a semi-quantitative or quantitative method to analyze substrates and materials [4,21,24]. Minerals from dental tissues are imperfect forms of hydroxyapatite, which result from the incorporation of “impure” ion crystals from tissue fluids as well as mineral crystals during hard tissue formation [4]. When dental mineral tissues are calcium-deficient, such as carbonated hydroxyapatite, they may contain ions, such as Na, K, Mg, Cl, Zn, Pb, Cu, and Al [4,34]. Hydroxyapatite ion exchange can exert a greater stress on enamel tissue, making it more susceptible to solubility [4]. Thus, it is possible to notice the presence of Na, Mg, Cl, and K; this is supported by the detection of minerals through the EDS analysis in the present study.

With regard to eroded enamel surfaces, lower Ca and P concentrations were found for all types of toothpastes and were associated with the dissolution of hydroxyapatite. Thus, the loss of Ca and P ions after the erosion–abrasion cycle indicates that the toothpaste type did not prevent the dissolution of hydroxyapatite in relation to the control surface [35]. Furthermore, Ca and P were more evident in enamel than in dentin, consistent with the findings of another study that investigated the chemical composition of eroded dental tissues and concluded that enamel naturally contains a higher concentration of these compounds [4]. NaF toothpaste seems to have a potential effect on ERMGIC because no differences in Ca and P were found between the control and eroded surfaces. Sn_2_ was detected in a few eroded dentin specimens. Although it is an anti-erosive toothpaste, in this study, it appeared to have obliterated the dentinal tubules (Figure 4F) and acted as a desensitizer. Some parts of the precipitates are loosely bound to the dentin surface and can be easily removed by brushing, which may reduce the protective effect. Lower tissue loss has been reportedly observed with toothpastes having lower pH values, higher fluoride concentrations, lower Ca and P concentrations, larger solid particles, and higher surface wettability [36]. Dentinal tubule occlusion has also been shown to be influenced by the presence of Sn^2+^ [36]. In the present study, the pH values of the toothpastes were as follows: WF, 5.59; NaF, 7.24; and SnF_2_, 6.62. These are considered high pH values. This may also have contributed to the lower protective effects of the toothpaste. In another study using EDS analysis, the efficacy of solutions containing SnF_2_ was related to the incorporation of Sn^2+^ ions into the mineralized dentin, when the organic portion was preserved on the subsurface [34]. However, Sn^2+^ precipitation occurs when the organic portion is removed from the surface [34]. Furthermore, higher Sn^2+^ concentrations are associated with higher fluoride concentrations (ppm) [34]. Another interesting finding was the presence of silica in the most eroded dentin surfaces with all toothpaste types. According to Ganss et al. [9], silica concentrations of up to 10% could be more harmful to surfaces than concentrations above this value. However, specific compositional information for the toothpastes studied was not provided by the manufacturers; this was a limitation of the present study.

With regard to the restorative materials, ionomeric materials seem to be influenced by the action of fluoride-based toothpastes, as the eroded RMGIC surfaces in the present study showed increased Ca and decreased F levels after brushing with NaF and SnF_2_. This was probably due to ion exchange with the environment, which may have been associated with the material’s ability to stabilize the pH and simultaneously allow fluoride release into the environment [33]. In addition, NaF and SnF_2_ resulted in greater alterations on eroded surfaces, which may be consistent with the findings of SEM analyses (Figure 4H,I), which showed greater changes in the material after the erosion–abrasion cycles. In contrast, CR showed a similar composition to Si and Zr (Figure 4J–L) after the erosion–abrasion cycles, corroborating the nanomechanical properties that also remained constant. Guler et al. [36] investigated the effects of beverages with different pH and citric acid levels on various resin-based restorative materials (CR and RMGIC) using atomic force microscopy and SEM analysis. They observed that the ionomeric material presented with deep cracks and spaces between particles, while the CR group showed no significant changes, consistent with the SEM findings in the present study. Fluoride-based toothpastes affected the structural composition of the ionomeric material, as observed on the SEM images (Figure 4H,I). On the other hand, CR showed no changes in the chemical composition or significant morphological surface alterations (Figure 4J–L).

Raman spectroscopy is an analytical technique capable of measuring the molecular composition and vibration of a substrate or material, and it provides information about chemical changes in specimens [23]. In dentistry, this tool is useful for analyzing calcium fluoride formation in the enamel and the resin–dentin interface in restored teeth [37]. Previous studies used phosphate (960 cm^−1^), which indicates the P–O stretch associated with hydroxyapatite [37]. Therefore, the analysis of the phosphate concentration within the enamel is a good indicator of the degree of mineralization [37]. In contrast, an in vitro study investigating caries lesions revealed that carious tissues treated with highly abrasive toothpastes (without brushing) showed a characteristic mineral distribution [11]. During an erosive process, phosphate release can be expected once the hydroxyapatite is dissolved. In addition, biological apatite is calcium-deficient and contains substantial amounts of carbonate (1070 cm^1^) [37]. The bands represent the intensity of the signal according to the frequency, and the mathematical exploitation of this allows for comparative and quantitative analyses. It is expected that phosphate is released during erosive processes, resulting in a decrease in the band intensity [37]. In the present study, there were no differences among the types of toothpastes used. However, the phosphate areas in the eroded enamel showed lower values than those in the control enamel brushed with the SnF^2^-based toothpaste. Carbonate areas in the eroded enamel presented lower values than those in the control enamel brushed with NaF. For other surfaces as well as teeth brushed with WF, no changes were found in phosphate and carbonate bands after erosion–abrasion cycles. One study revealed no differences between intact and eroded enamel in extracted primary teeth [38].

Furthermore, because the volumes involved were small, the amount of phosphate released from the apatite crystals may have been overestimated [38]. For dentin surfaces, there were differences between the eroded and control surfaces, as phosphate and carbonate decreases were observed after brushing with all types of toothpastes. In other words, dentin surfaces are affected by not only the toothpastes but also the erosion–abrasion cycles.

This study has some limitations. The Poisson’s ratio was not calculated. Another limitation is that the area of analysis with nanoindentation was quite small, and the property of this area may not reflect that of the material as a whole. Further in situ and in vivo studies are required for the thorough analysis of the mechanical and chemical alterations in CR and RMGIC restorations in eroded enamel and dentin, because the presence of saliva and the salivary pellicle influences the dissolution and abrasive behavior of dental substrates and restorative materials as well as the formation and stability of fluoride precipitates.

In conclusion, the fluoride-containing toothpastes were capable of preserving the main chemical components of the dentin adjacent to the restorative materials under erosive–abrasive conditions.

## Figures and Tables

**Table 1:** Toothpastes and restorative materials used in this study.

Material	Application Mode	Composition	Manufacturer
Adper Single Bond 2 (Adhesive system)	Apply one layer of adhesive, wait for 20 s, air stream for 5 s, and polymerize for 10 s	Bis-GMA, HEMA, dimethacrylates, ethanol, water, a novel photoinitiator system and a methacrylate functional copolymer of polyacrylic and polyitaconic acids	3M ESPE, St. Paul, MN, USA
Filtek Z350 XT (color A2B) Batch: 672,912	Apply increments of 2 mm and polymerize for 20 s each	Bis-GMA, UDMA, Bis-EMA, TEGDMA, PEGDMA, Zirconia and agglomerates of silica and camphorquinone	3M ESPE, St. Paul, MN, USA
Fuji II LC (color A3) Batch: 17,051,316	Apply GC conditioner for 20 s and rinse and dry for 10 s. Dispense one level scoop of powder with two drops of liquid and mix for 15–20 s. Transfer the mixture to the centrix syringe and polymerize for 40 s.	Powder: fluor-amino-silicate glass. Liquid: aqueous solution of polycarboxylic acid, TEGDMA and HEMA	GC, Tokyo, Japan
Curaprox Enzycal Zero (RDA-60) * Batch: 442MHDEXP1121	Fluoride-free toothpaste (WF)	Water, sorbitol, hydrated silica, Glycerin, Steareth-20, titanium dioxide (Cl 77,891), flavor, sodium phosphate, carrageenan, sodium chloride, citric acid, sodium benzoate, potassium thiocyanate, glucose oxidase, amyloglucosidase, lactoperoxidase	Trybol, Neuhausen am Rheinfall, Swiss
Colgate Total 12 (RDA-70/80) * Batch: 6184BR121R	Sodium Fluoride Toothpaste (NaF)	Sodium fluoride (1450 ppm as NaF) water, triclosan, sorbitol, silica, sodium lauryl sulfate, PMV/MA copolymer, sodium hydroxide, saccharin sodium, titanium dioxide	Colgate-Palmolive, São Bernardo do Campo, SP, Brazil.
Crest Pro-Health (RDA-155) * Batch: 6039GF	Stannous Fluoride Toothpaste (SnF_2_)	Stannous fluoride (1100 ppm F as SnF_2_) glycerin, hydrated silica, sodium hexametaphosphate, propylene glycol, peg 6, water, zinc lactate, trisodium phosphate, sodium lauryl sulfate, carrageenan, sodium saccharin, xanthan gum, blue 1	Procter & Gamble, Cincinnati, OH, USA

* RDA values according to manufacturers.

**Table 2:** Nanohardness values for dental surfaces and restorative materials treated with different toothpastes. Mean (SD) H values expressed in GPa.

Factors	ERMGIC-C	ECR-C	RMGIC-C	CR-C	DRMGIC-C	DCR-C
**WF**	2.97 (0.45) ^Aa^	2.66 (0.40) ^Ab^	0.47 (0.20) ^Ab^	0.69 (0.12) ^Aa^	0.68 (0.15) ^Aa^	0.63 (0.10) ^Aa^
**NaF**	2.89 (0.73) ^Aa^	2.96 (0.43) ^Aa^	0.41 (0.19) ^Ab^	0.67 (0.17) ^Aa^	0.59 (0.12) ^Ba^	0.61 (0.15) ^Aa^
**SnF_2_**	3.09 (0.83) ^Aa^	2.98 (0.63) ^Aa^	0.49 (0.21) ^Ab^	0.70 (0.21) ^Aa^	0.65 (0.13) ^Aba^	0.67 (0.15) ^Aa^
**Factors**	**ERMGIC-E**	**ECR-E**	**RMGIC-E**	**CR-E**	**DRMGIC-E**	**DCR-E**
**WF**	0.51 (0.17) ^Aa^ *	0.55 (0.22) ^Aa^ *	0.29 (0.09) ^Ab^ *	0.64 (0.08) ^Aa^	0.05 (0.02) ^Aa^ *	0.10 (0.05) ^Aa^ *
**NaF**	0.52 (0.24) ^Aa^ *	0.50 (0.30) ^Aa^ *	0.34 (0.16) ^Ab^	0.65 (0.18) ^Aa^	0.08 (0.04) ^Aa^ *	0.06 (0.02) ^Aa^ *
**SnF**_2_	0.27 (0.07) ^Aa^ *	0.23 (0.06) ^Aa^ *	0.25 (0.14) ^Ab^ *	0.63 (0.11) ^Aa^	0.08 (0.03) ^Aa^ *	0.07 (0.02) ^Aa^ *

Uppercase letters compare toothpastes for each control and eroded side. Lowercase letters compare the surfaces separately (*p* < 0.05). * Significant difference between the control and eroded surfaces. SD, standard deviation; H, nanohardness; GPa, gigapascal.

**Table 3:** Mean (SD) calcium/phosphorus ratios for the enamel surfaces determined by EDS analysis.

Factors	ERMGIC-C	ERMGIC-E	ECR-C	ECR-E
**WF**	1.80 (0.10) ^Aa^	1.78 (0.12) ^Aa^	1.79 (0.10) ^Aa^	1.80 (0.12) ^Aa^
**NaF**	1.75 (0.14) ^Aa^	1.78 (0.16) ^Aa^	1.80 (0.10) ^Aa^	1.87 (0.08) ^Aa^
**SnF_2_**	1.81 (0.02) ^Aa^	1.71 (0.09) ^Aa^	1.75 (0.12) ^Aa^	1.77 (0.09) ^Aa^

Uppercase letters compare toothpastes for each surface. Lowercase letters compare surfaces for each toothpaste (*p* < 0.05). SD, standard deviation.

**Table 4:** Mean (SD) calcium/phosphorus ratios for the dentin surfaces determined by EDS analysis.

Factors	DRMGIC-C	DRMGIC-E	DCR-C	DCR-E
**WF**	1.74 (0.08) ^Aa^	0.53 (0.83) ^Ab^	1.71 (0.09) ^Aa^	0.62 (0.96) ^Bb^
**NaF**	1.68 (0.08) ^Aa^	1.12 (0.87) ^Aa^	1.95 (0.35) ^Aa^	1.77 (0.12) ^Aa^
**SnF_2_**	1.70 (0.08) ^Aa^	0.53 (0.81) ^Aa^	1.74 (0.10) ^Aa^	1.22 (0.95) ^ABab^

Uppercase letters compare toothpastes for each surface. Lowercase letters compare surfaces for each toothpaste (*p* < 0.05). SD, standard deviation.

**Table 5:** Mean (SD) carbonate/phosphate ratios for the enamel surfaces determined by Raman analysis (200 × 200 µm).

Factors	ERMGIC-C	ERMGIC-E	ECR-C	ECR-E
**WF**	0.06 (0.08) ^Aa^	0.04 (0.01) ^Aa^	0.04 (0.01) ^Aa^	0.04 (0.01) ^Aa^
**NaF**	0.05 (0.01) ^Aa^	0.05 (0.02) ^Aa^	0.03 (0.01) ^Aa^	0.04 (0.01) ^Aa^
**SnF_2_**	0.04 (0.02) ^Aa^	0.08 (0.13) ^Aa^	0.04 (0.01) ^Aa^	0.04 (0.02) ^Aa^

Uppercase letters compare toothpastes for each surface. Lowercase letters compare surfaces for each toothpaste (*p* < 0.05). SD, standard deviation.

**Table 6:** Mean (SD) carbonate/phosphate ratios in the enamel surfaces by Raman analysis (200 × 200 µm).

Factors	DRMGIC-C	DRMGIC-E	DCR-C	DCR-E
**WF**	0.33 (0.20) ^Bb^	0.35 (0.05) ^Aab^	0.42 (0.07) ^Aa^	0.29 (0.04) ^Ab^
**NaF**	0.42 (0.06) ^Aa^	0.36 (0.08) ^Aab^	0.39 (0.04) ^Aa^	0.26 (0.08) ^Ab^
**SnF_2_**	0.45 (0.07) ^Aa^	0.31 (0.08) ^Ab^	0.42 (0.06) ^Aa^	0.25 (0.09) ^Ab^

Uppercase letters compare toothpastes for each surface. Lowercase letters compare surfaces for each toothpaste (*p* < 0.05). SD, standard deviation.

## Data Availability

The data presented in this study are available on request from the corresponding author.
